# The Eye and the Brain: Photonic Devices in Neuro-Ophthalmology

**DOI:** 10.3390/diseases14060207

**Published:** 2026-06-10

**Authors:** Alessandro Avitabile, Marco Zeppieri, Ludovica Cannizzaro, Giuseppe Gagliano, Maria Francesca Cordeiro, Fabiana D’Esposito, Francesco Cappellani, Maria Vadalà, Vincenza Maria Elena Bonfiglio

**Affiliations:** 1 Biomedicine, Neuroscience and Advance Diagnostic (BIND) Department, University of Palermo, 90128 Palermo, Italy; alessandro.avitabile@unipa.it (A.A.);; 2 Department of Ophthalmology, University Hospital of Udine, 33100 Udine, Italy; 3 Department of Medicine, Surgery and Health Sciences, University of Trieste, 34129 Trieste, Italy; 4 Eye Clinic, Catania University, Policlinico G. Rodolico, 95121 Catania, Italy; 5 Imperial College Ophthalmic Research Group (ICORG) Unit, Imperial College, 153-173 Marylebone Rd, London NW1 5QH, UK; 6 Department of Medicine and Surgery, University of Enna “Kore”, Piazza dell’Università, 94100 Enna, Italy; 7 Mediterranean Foundation “G.B. Morgagni”, 95125 Catania, Italy

**Keywords:** optical coherence tomography, photonic imaging, neuro-ophthalmology, retinal neurodegeneration, optic neuritis, multiple sclerosis, glaucoma, detection of apoptosing retinal cells, two-photon microscopy, neurodegenerative biomarkers

## Abstract

Photonic imaging technologies have profoundly transformed neuro-ophthalmic diagnostics by enabling non-invasive visualization of neurodegenerative processes at the retinal level. This review examines how advanced light-based modalities provide unprecedented insights into the structural, physiologic, and biologic relationships between the eye and brain in conditions such as optic neuritis, multiple sclerosis, and glaucoma. Optical coherence tomography has emerged as an essential tool for quantifying thinning of the retinal nerve fiber layer and ganglion cell layer, serving as reliable biomarkers of axonal loss and disease progression across multiple sclerosis subtypes and optic neuropathies. Detection of apoptosing retinal cells imaging enables real-time visualization of retinal ganglion cell apoptosis preceding irreversible structural damage, offering a critical window for early intervention in various neurodegenerative conditions, in particular, glaucoma. Two-photon microscopy with adaptive optics enables subcellular-resolution imaging of retinal neurons, microvascular dynamics, and inflammatory processes in vivo, facilitating the characterization of neurodegenerative mechanisms at unprecedented spatial scales and redefining neuro-ophthalmology by positioning the retina as an accessible extension of the central nervous system. This review critically examines how established and investigational photonic imaging modalities may support earlier disease detection, longitudinal monitoring, and biomarker development in neuro-ophthalmic and neurodegenerative disorders, with potential implications for more timely and targeted management strategies.

## 1. Introduction

Neuro-ophthalmology bridges neurology and ophthalmology by focusing on the eye–brain axis. As an accessible extension of the central nervous system, the retina provides a unique, non-invasive window into neurodegenerative processes in the brain [1]. This concept has gained momentum with the rapid advancement of photonic imaging technologies, providing remarkable detail about retinal structure and function [2]. The broad significance is clear: if subtle retinal alterations can mirror pathological processes in diseases like multiple sclerosis (MS), glaucoma, and optic neuritis, then ophthalmic imaging could offer an invaluable early indicator of brain health. By enabling clinicians and researchers to visualize neurodegeneration in the eye directly, photonic imaging technologies offer increasingly valuable opportunities for earlier diagnosis and improved understanding of neurological disease. In this review, OCT and OCT-A are treated as established clinical modalities whose ongoing technological evolution continues to expand their translational relevance in neuro-ophthalmology. By contrast, DARC, adaptive optics, two-photon imaging, hyperspectral retinal imaging, and fluorescence lifetime imaging ophthalmoscopy (FLIO) are discussed as investigational or early translational approaches that may extend retinal imaging beyond conventional structural assessment. This distinction aids in matching the review’s scope to the evidence’s current level of maturity.

**Figure 1:**
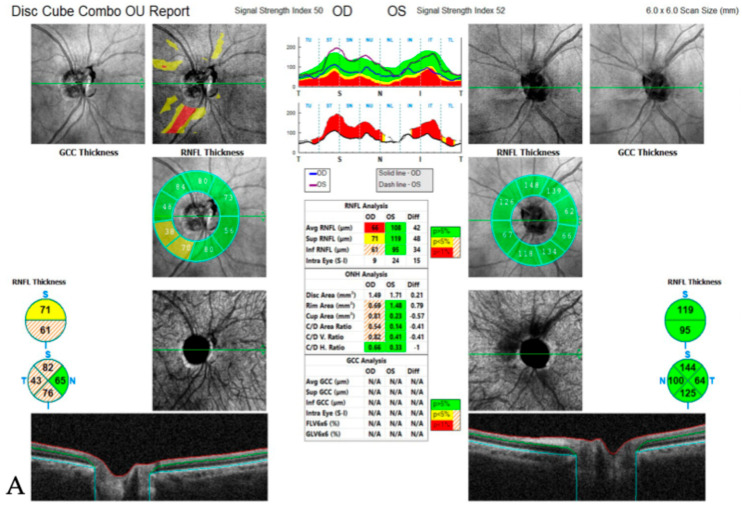

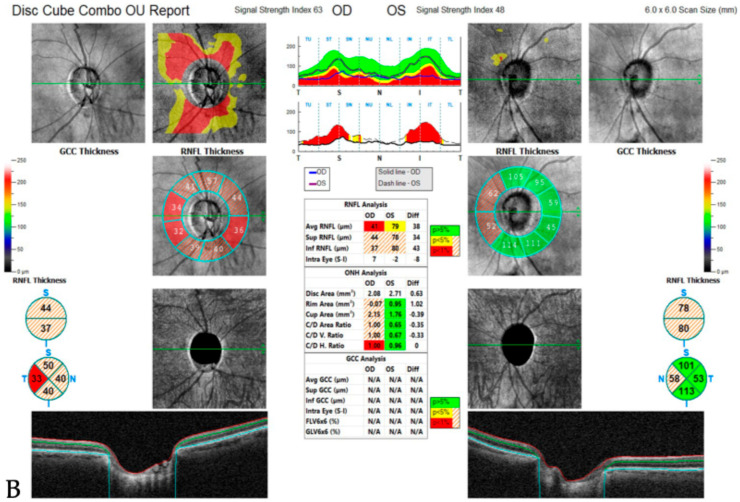
Representative OCT examples of peripapillary RNFL loss. Representative disc cube OCT outputs illustrating retinal nerve fiber layer (RNFL) assessment in both eyes. Image (**A**) shows an asymmetric pattern of RNFL thinning, with greater structural involvement in the right eye and relative preservation of the fellow eye. Image (**B**) shows a more advanced and diffuse pattern of RNFL loss, again more pronounced in the right eye. Sectoral thickness maps, circumpapillary profiles, and cross-sectional B-scans highlight how OCT can provide a topographic and quantitative evaluation of neuroaxonal damage. Representative OCT image acquired using the SOLIX optical coherence tomography system (Optovue Inc., Fremont, CA, USA).

Optical coherence tomography (OCT) represents a major milestone in biomedical optics, enabling depth-resolved, cross-sectional visualization of tissue microstructure via low-coherence interferometry. Since its first experimental validation, OCT has been widely regarded as an “optical biopsy” modality, capable of generating micrometer-scale axial sections within scattering tissues. Its clinical translation was particularly rapid in ophthalmology, where the transparency of the ocular media supports stable acquisition and high-quality delineation of the retinal layers. Since the initial report, OCT has progressed from a proof-of-concept imaging technique to a clinically essential platform that facilitates quantitative and longitudinal evaluation of neural and vascular structures in the retina and optic nerve head, thereby aligning seamlessly with neuro-ophthalmology’s requirement for objective biomarkers at the eye–brain interface. Modern OCT systems have achieved significant performance enhancements that have transformed measurement capabilities in both everyday practice and research, notably through accelerated acquisition speeds, improved contrast mechanisms, and the integration of multimodal imaging. Contemporary implementations have facilitated higher-density sampling, enhanced layer segmentation, and integration with complementary contrasts, collectively improving the interpretability of retinal structural metrics and enabling physiologic and vascular extensions, such as angiographic assessments of the microvasculature. These advancements have established a technical basis for the clinical development of OCT and its continuous extension into neuro-ophthalmic applications that necessitate sensitivity to nuanced changes over time [3]. Simultaneously, resolution has remained a pivotal catalyst for innovation, leading to the development of OCT-derived microscopy techniques to optimize tissue-level detail when traditional configurations have proven inadequate for specific biological inquiries. Representative engineering developments in OCT-derived microscopy and handheld implementation further illustrate the technical trajectory of these platforms and their broader translational potential [4]. The development of photonic imaging modalities described in this review has been shaped by many key investigations. A first report by Huang et al. demonstrated the feasibility of optical coherence tomography as a depth-resolved, non-invasive technique to acquire cross-sectional images of biological tissues, opening the possibility of its application in retinal imaging and neuro-ophthalmology [5]. Petzold et al. presented a comprehensive synthesis of evidence in multiple sclerosis demonstrating that retinal layer segmentation, particularly the pRNFL and ganglion cell–inner plexiform layer measurements, can be used as reliable markers of neuroaxonal injury and disease-related retinal atrophy [6]. A major breakthrough in OCT angiography is the split-spectrum amplitude-decorrelation angiography method proposed by Jia et al., which enabled non-invasive imaging of retinal microvascular perfusion [7]. Similarly, the research on adaptive optics by Liang, Williams and Miller found that the correction of ocular aberrations could substantially improve the quality of the retinal images and allow high-resolution in vivo observation of the retinal structures [8]. Together, these contributions provide the technological foundation for many of the clinical and translational imaging modalities described in this article. OCT exemplifies how photonic tools have transformed neuro-ophthalmic practice. OCT provides high-resolution cross-sectional images of the retina, allowing quantitative measurement of the retinal nerve fiber layer (RNFL) and ganglion cell layer thickness. Representative OCT acquisition and peripapillary RNFL outputs are shown in Figure 1. These examples illustrate how B-scan morphology, sectoral thickness maps, and normative comparison plots can be integrated to distinguish preserved from abnormal retinal nerve fiber layer profiles. In MS and its common manifestation, optic neuritis, OCT studies first established that RNFL thinning correlates with visual dysfunction and axonal loss in the optic nerve [9,10]. Longitudinal data confirm that progressive retinal layer thinning in MS patients is associated with disease activity and disability progression—for instance, thinning after an optic neuritis episode is linked to a higher risk of future MS relapses [11,12]. Such findings firmly position OCT-derived metrics as biomarkers of neurodegeneration in MS, reflective of broader CNS atrophy. Indeed, current research and reviews endorse OCT (and its angiographic variant, OCT-A) as valuable, objective measures for monitoring inflammation and neurodegeneration in MS [13,14]. Beyond MS, similar applications of OCT extend to other neurologic disorders, including in pediatric populations, underscoring its broad relevance [15,16]. Overall, OCT has become an indispensable tool in neuro-ophthalmology for translating retinal changes into neurologically meaningful information.

Glaucoma, though primarily an ocular disease, is increasingly recognized as a chronic neurodegeneration of retinal ganglion cells with brain involvement. Here too, photonic imaging is pivotal. Standard OCT measurements of the ganglion cell complex and RNFL are routine for early glaucoma detection and tracking, as thinning of these layers reflects the loss of retinal ganglion cells [17]. However, purely structural metrics lag behind the actual disease process, since significant cell death may occur before gross thinning is apparent. To address this, the Detection of Apoptosing Retinal Cells (DARC) technique was developed as a novel imaging approach to visualize neuronal apoptosis in vivo [18]. DARC uses a fluorescent marker that binds to apoptotic retinal cells, which can then be seen as bright spots on a confocal scanning laser ophthalmoscope [19]. Remarkably, clinical studies have demonstrated real-time imaging of single retinal ganglion cells undergoing apoptosis in patients with glaucoma using this method [18]. By directly capturing neural death as it happens, DARC offers a critical window for intervention, potentially alerting clinicians to glaucoma activity well before irreversible vision loss ensues. While DARC remains investigational, its emergence highlights how cellular photonic imaging, targeting apoptotic events rather than anatomy alone, can augment our diagnostic arsenal for neuro-ophthalmic conditions.

Pushing the frontiers of resolution and insight further, researchers have turned to two-photon microscopy combined with adaptive optics (AO) to study the eye–brain connection at the cellular and molecular level. Two-photon excitation fluorescence microscopy involves near-infrared pulsed laser light that can penetrate the eye and excite fluorescent signals from deep retinal cells with minimal damage. When augmented with adaptive optics, a technique that corrects optical aberrations of the eye, this approach achieves near diffraction-limited imaging in the living retina [20]. For the first time, it has become possible to visualize subcellular structures and dynamic processes in retinal neurons and glia in vivo. For example, AO-assisted two-photon imaging in mouse models has revealed fine details of retinal ganglion cell dendrites and real-time microvascular blood flow that were previously beyond reach [20,21].

Pioneering studies have even used two-photon microscopy to observe immune cell behavior in retinal disease: in diabetic mice, investigators directly tracked hyperactive microglia surveying the retina long before any vascular damage was clinically evident [22]. Such findings provide a vivid illustration of how high-resolution optical imaging can uncover early neuroinflammatory changes, offering clues to pathology that might parallel changes in the brain. Notably, two-photon retinal imaging is not confined to animal models, and recent work has demonstrated the feasibility and safety of two-photon excited scanning laser ophthalmoscopy in human volunteers, opening the door to clinical applications of this technology; however, these studies have not yet attained the cellular-level resolution observed in mouse retinal imaging [23,24]. These advances are redefining the limits of neuro-ophthalmic imaging and reinforce the view of the retina as an approachable outpost of the brain’s neural network.

In addition to OCT and two-photon techniques, a host of other emerging photonic tools are enriching the field. Hyperspectral retinal imaging, for instance, can detect metabolic and structural changes by measuring spectral signatures of the retina and has been applied to identify retinal biomarkers in neurodegenerative diseases such as Parkinson’s disease [25]. Fluorescence lifetime imaging ophthalmoscopy (FLIO) provides contrast based on the decay time of natural fluorophores in retinal tissue. It has shown promise in linking retinal metabolic abnormalities to cortical pathology in disorders that affect vision [26]. There is also growing interest in multimodal retinal imaging platforms; for example, combinations of photoacoustic imaging and OCT have demonstrated the feasibility of integrating microvascular and oxygenation-sensitive retinal imaging in vivo in preclinical settings, illustrating the broader technological direction of multimodal retinal imaging [27].

The field is experiencing a phase of rapid expansion and accelerating innovation, with photonic imaging modalities yielding a wealth of data on the eye–brain axis. Nevertheless, translating these innovations into clinical practice brings challenges and open questions. One area of debate is how directly retinal changes reflect central nervous system processes in each condition. While many studies support a strong correspondence, others urge caution, noting that retinal pathology might not always mirror brain pathology in a one-to-one manner [2]. Such divergences in findings underscore the need for further longitudinal research and standardization of imaging biomarkers. Despite these challenges, a unifying theme is emerging: advanced optical imaging is enabling investigators to probe neurodegeneration non-invasively and repeatedly, in ways that were unimaginable a decade ago.

Considering these developments, this review highlights the most recent advances in photonic imaging and examines how they are transforming neuro-ophthalmology. We focus on how technologies such as OCT, DARC, adaptive optics and two-photon microscopy are being leveraged to better understand and diagnose neuro-ophthalmic diseases, including MS, optic neuritis, and glaucoma. By reviewing the current state of research—and noting both breakthroughs and remaining controversies—we aim to make this rapidly evolving field accessible to scientists and clinicians outside ophthalmology. What this review adds is the translational perspective in an integrated manner. Rather than focusing on one imaging modality or one neurodegenerative condition, this article compares established and investigational photonic technologies across the broader neuro-ophthalmologic spectrum. We distinguish between clinically mature tools such as OCT and OCT-A and early translational or experimental approaches, including DARC, adaptive optics, two-photon imaging, FLIO, hyperspectral imaging and multimodal photoacoustic platforms. It also reviews the biological targets and translational maturity of each modality, including structural neuroaxonal loss, retinal microvascular change, apoptotic activity, metabolic dysfunction, and cellular-level retinal dynamics. Finally, we stress interpretive caveats, specifically the unresolved issue of whether retinal imaging abnormalities reflect primary disease mechanisms, secondary effects of neuronal loss, or associative biomarkers of overall central nervous system pathology.

## 2. Materials and Methods

This review is a narrative literature review that provides a comprehensive synthesis of existing evidence on photonic retinal imaging in neuro-ophthalmology. This study did not require institutional ethical approval as it did not involve new experiments or patient data.

### 2.1. Literature Search Strategy

To locate papers relevant to the topic at hand, we conducted an exhaustive literature search across the most prominent scientific databases. Specifically, we examined PubMed (MEDLINE), Embase, and Web of Science literature. We focused on research conducted within the past ten to fifteen years to consider the most current technical advancements. However, no strict start date was applied. The search was last updated in December 2025 to include the most recent studies. As this was a narrative literature review, no laboratory experiments, pharmacological agents, reagents, or dedicated statistical software were used. The OCT images included in the manuscript were used only for illustrative purposes and were not part of a prospective experimental protocol or quantitative image-analysis workflow. When applicable, the imaging device and software details are provided in the corresponding figure legend.

We developed our search queries using several combinations of terms that were associated with neuro-ophthalmologic illnesses and photonic or light-based retinal imaging modalities. For example, key imaging modality terms included: “optical coherence tomography (OCT),” “adaptive optics retinal imaging,” “two-photon microscopy,” and “detection of apoptosing retinal cells (DARC)”. We combined several disease-related phrases, including “multiple sclerosis (MS),” “optic neuritis,” “glaucoma,” “Alzheimer’s disease,” and “Parkinson’s disease,” to narrow the search results to neuro-ophthalmic settings. To ensure that retrieved papers specifically linked the imaging modalities with neurodegenerative or neuro-ophthalmic disorders, we used Boolean operators (AND/OR).

We restricted our search to English-language articles, and the final analysis incorporated only studies published in peer-reviewed journals. In addition, the reference lists of key articles and recent review papers were manually examined to identify any relevant studies that the database queries might have missed. Conference abstracts, meeting proceedings, and non-peer-reviewed conference materials were excluded from our literature search.

### 2.2. Eligibility Criteria

Prior to screening the search results, we established clear inclusion and exclusion criteria to guide the selection of literature and ensure that only relevant, high-quality sources were included.

Inclusion criteria: We included publications that met all of the following conditions:

▪ Published in a peer-reviewed journal and available in full-text English.
▪ Investigated or discussed photonic or light-based ocular imaging techniques (such as OCT, OCT angiography, DARC, two-photon microscopy, adaptive optics imaging, fluorescence lifetime imaging ophthalmoscopy, hyperspectral retinal imaging, or similar modalities).
▪ Focused on the application of these imaging modalities in the context of neurodegenerative or neuro-ophthalmologic diseases (e.g., multiple sclerosis (MS), optic neuritis, glaucoma, or related conditions where retinal changes reflect central nervous system pathology).
▪ Reported on the diagnostic value, monitoring utility, or significant findings linking retinal imaging results to neurological disease status or progression. This included both clinical studies and relevant translational or preclinical research providing insight into human disease mechanisms.

Exclusion criteria: We excluded articles if they met any of the following conditions:

▪ Not written in English or not published in a peer-reviewed scientific source.
▪ Did not specifically relate retinal imaging to neurological or neurodegenerative disease (for instance, studies focused solely on general ophthalmic conditions without addressing any central nervous system implications, or papers on photonic imaging technology that lacked a disease context).
▪ Consisted of single-patient case reports, very small case series (typically fewer than five subjects), or opinion pieces without new data, as well as purely technical engineering papers without translational, biological, or contextual relevance to neuro-ophthalmology.

Following the execution of the searches, all obtained titles and abstracts were evaluated for relevance based on these criteria. This screening was conducted by two authors independently to reduce selection bias. Citations that failed to meet the inclusion criteria were excluded at this stage. We acquired and examined the full-text articles to identify references potentially relevant to the final inclusion decisions. Disagreements between the two reviewers concerning eligibility were addressed through discussion and consensus. A total of 55 publications meeting the inclusion criteria were identified and included in the review. The narrative synthesis primarily emphasized studies with direct neuro-ophthalmic or neurodegenerative relevance and stronger clinical or translational value, while a limited number of contextual technical references were retained to frame the development or multimodal capabilities of specific imaging platforms.

### 2.3. Extraction and Synthesis of Data

We extracted key information from each of the 55 included sources, focusing on study design, subject population or model system (if applicable), photonic imaging modality used, and main findings related to the connection between ocular measures and neurological disease. We also identified key data elements, such as the methods and metrics used in each study, to evaluate the retina or optic nerve. Examples include structural measures such as retinal nerve fiber layer thickness assessed via OCT, detection of apoptotic retinal cells using DARC imaging, and cellular-level observations facilitated by adaptive optics. We documented reported correlations between the ocular findings and clinical indicators of neurological disease, including disability scores in multiple sclerosis, visual field loss in glaucoma, and relevant brain imaging measures.

We organized the presentation of results by major imaging modality and disease context to illustrate each photonic tool’s contribution to understanding the eye–brain connection. We assembled evidence from OCT and OCT angiography to show that retinal layer measurements and microvascular alterations serve as biomarkers in conditions such as MS and glaucoma. We reviewed studies utilizing DARC to highlight its ability to detect retinal cell apoptosis as an early indicator of neurodegenerative damage. Adaptive optics imaging enabled ultra-high-resolution visualization of retinal neurons and microvasculature in neuro-ophthalmic disorders. Results from two-photon microscopy studies (mainly in experimental models) were also examined to elucidate cellular-level neuro-glial interactions in the retina that reflect central nervous system pathology.

Our review also considered investigational techniques such as hyperspectral retinal imaging and fluorescence lifetime imaging ophthalmoscopy (FLIO), while taking into account their current translational maturity and evidentiary limitations. As a narrative review, this synthesis did not aim to provide a purely descriptive summary; instead, the included studies were compared critically to identify consistent findings, methodological strengths, potential sources of bias, areas of disagreement, and remaining knowledge gaps. Greater interpretive weight was assigned to larger cohorts, meta-analyses, well-controlled clinical studies, and robust translational studies, whereas findings from small exploratory studies were interpreted more cautiously.

## 3. Results

The eye has long been viewed as a “window to the brain,” given that the retina is a developmental extension of the central nervous system (CNS) [1,28]. Consistent with this, many CNS neurodegenerative conditions produce observable retinal abnormalities, and in some cases, ocular symptoms even precede the conventional diagnosis of the neurological disease [1,28]. Advanced retinal imaging has revealed structural and vascular changes that correspond with broader neurodegenerative mechanisms across various disorders, including multiple sclerosis, Alzheimer’s disease, Parkinson’s disease, and glaucoma [28,29,30]. Below, we summarize key retinal imaging findings in these conditions, drawing on the evidence from the selected studies.

### 3.1. Retinal Imaging Findings in Multiple Sclerosis (MS)

In MS, optical coherence tomography (OCT) has emerged as a valuable tool for detecting inflammation and neuroaxonal loss in the visual pathway [9,10,11,12,13,14,15,16,31]. OCT studies consistently show that MS patients have significant thinning of the peripapillary retinal nerve fiber layer (pRNFL) and macular ganglion cell/inner plexiform layer (GCIPL) compared to healthy controls [9,12,31]. These structural retinal measures correlate strongly with clinical parameters of MS. For example, reduced RNFL thickness is associated with longer disease duration and higher disability (EDSS) scores, reflecting cumulative axonal degeneration over time [9,11,12,31]. Thinning of the ganglion cell layer also correlates with worse visual function and can predict overall axonal damage in patients with MS [14,15,31]. Notably, OCT metrics not only reflect past damage but also have prognostic value. A recent meta-analysis of longitudinal studies (including over 3600 MS patients) identified specific OCT thresholds that predict future disease progression: a baseline pRNFL thickness ≤ 88 μm or GCIPL ≤ 77 μm was linked to a more than two-fold increased risk of disability worsening, and accelerated annual thinning of these layers further increased this risk [31]. In other words, MS patients with very thin retinal layers or faster retinal atrophy are significantly more likely to experience subsequent physical and cognitive decline [31]. These findings highlight retinal layer thickness as a quantifiable biomarker for MS progression [13,14,16,31].

In addition to structural changes, retinal microvascular alterations have been observed in MS using OCT-angiography (OCT-A) [13,14,32]. MS eyes show a reduction in vessel density within the superficial capillary plexus of the retina compared to controls [13,32]. This microvascular loss appears to parallel neurodegeneration, as lower superficial vascular density has been associated with greater disability in MS [13,14].

Similar OCT-A changes have also been reported in eyes without a documented history of optic neuritis; however, this finding should be interpreted cautiously, because the absence of clinically recognized optic neuritis does not exclude subclinical optic pathway involvement in MS [9,10,12,13]. Therefore, reduced retinal perfusion and vessel density in MS should not be interpreted automatically as evidence of primary retinal vascular pathology, as these changes may also reflect secondary vascular remodeling related to ganglion cell and axonal loss, reduced metabolic demand, or chronic subclinical visual pathway damage [13,14,31,32]. Deep capillary plexus changes are less pronounced, presumably because MS primarily causes atrophy of inner retinal layers (nourished by the superficial plexus) while sparing deeper layers [13,14]. Indeed, multiple studies have confirmed that retinal perfusion is significantly reduced in MS patients regardless of optic neuritis history [13,14,16,32]. The interpretation of retinal microvascular abnormalities in multiple sclerosis remains challenging because OCT-A findings do not, by themselves, establish whether vascular alterations are primary disease-related events or secondary consequences of neuroaxonal degeneration [13,14,32]. Reduced retinal perfusion and vessel density may reflect reduced metabolic demand following ganglion cell and axonal loss rather than an isolated microvascular disorder [9,12,31]. This distinction is crucial because subclinical optic neuropathy can occur in multiple sclerosis even in the absence of a recorded episode of acute optic neuritis [9,10,12]. Therefore, OCT-A abnormalities in clinically asymptomatic eyes should not be interpreted automatically as evidence of primary retinal vascular pathology. Evidence from optic nerve injury models and clinical optic neuropathies indicates that neuronal damage can itself induce subsequent retinal vascular remodeling. Accordingly, current OCT-A findings in multiple sclerosis should be regarded as associative biomarkers of disease burden, while the extent to which they represent primary vascular involvement rather than secondary neurodegenerative change remains unresolved [13,14,32].

Overall, the MS literature indicates that retinal OCT and OCT-A can detect subclinical damage, including thinning of inner retinal layers and loss of retinal microvasculature, both of which correlate with MS disease burden [9,14,31]. These ocular biomarkers are being investigated as easily accessible indicators of neurodegeneration in MS that might aid in monitoring progression or treatment response [13,14,31].

### 3.2. Retinal Imaging Findings in Alzheimer’s Disease (AD)

Patients with Alzheimer’s disease exhibit distinct retinal abnormalities that align with the widespread neurodegeneration characteristic of AD [2,33,34]. Histopathological and imaging studies show that AD’s hallmark proteins and neurodegenerative changes are present in the retina [2,33,34]. For instance, postmortem analyses have identified amyloid-beta (Aβ) plaques in the retinas of AD patients, particularly clustered in peripheral retinal regions that also show neuronal loss [33,34]. Parallel OCT imaging studies in living patients have demonstrated substantial loss of retinal ganglion cells and thinning of the RNFL in AD, even at early stages of cognitive impairment [33,34]. Such retinal thinning in AD often accompanies visual disturbances (e.g., narrowed visual fields, reduced contrast sensitivity) that AD patients experience [33,34]. Notably, retinal nerve fiber layer thinning and ganglion cell loss have been observed in mild cognitive impairment (MCI) cases as well, suggesting these changes arise early in the disease process [33,34]. Evidence increasingly indicates retinal neurodegenerative alterations in Alzheimer’s disease, identifiable through advanced imaging techniques even when clinical optic neuropathy is not apparent [2,33,34]. Advanced ocular imaging modalities have uncovered subtle retinal changes that would be invisible on a normal fundoscopic exam [2,33,34]. Advanced imaging has demonstrated retinal layer atrophy and diminished capillary density in Alzheimer’s disease, as reported by OCT and OCTA studies, underscoring retinal involvement associated with the condition. However, comorbid ocular diseases (e.g., glaucoma) represent important confounders when interpreting retinal thinning and perfusion metrics and should be carefully screened for or adjusted in analyses [33,34,35]. Some studies have reported reduced retinal vessel density and perfusion in AD, findings that align with evidence of cerebral microvascular dysfunction in this dementia [32,35]. However, such decreases should be interpreted cautiously, as they may reflect a secondary reduction in metabolic demand driven by neuronal loss rather than a primary microvascular deficit. Furthermore, novel imaging of retinal metabolism has provided metabolic evidence of AD’s impact on the eye. Fluorescence lifetime imaging ophthalmoscopy (FLIO), which measures the nanosecond-scale decay of natural retinal fluorophores, has shown altered metabolic signatures in AD retinas [26]. Because FLIO signals are linked to mitochondrial activity, these changes likely reflect the mitochondrial dysfunction and oxidative stress known to occur in AD [26]. In preliminary FLIO data, patients with AD (and similarly PD, as well as progressive MS) demonstrated prolonged retinal fluorophore lifetimes correlating with disease duration [26]. Such findings suggest that retinal metabolism is perturbed in AD, aligning with brain pathology, and support the concept that the retina can mirror AD-related biochemical changes [2,26,33]. In summary, AD is associated with a constellation of retinal changes—from Aβ plaque deposits and retinal thinning to microvascular and metabolic abnormalities—reinforcing the notion that retinal examinations could aid early AD detection or monitoring [2,29,33,34,36].

### 3.3. Retinal Imaging Findings in Parkinson’s Disease (PD)

Parkinson’s disease, like AD, produces measurable retinal alterations despite being primarily a movement disorder originating in the brain [2,37]. OCT studies have reported thinning of the peripapillary RNFL in patients with PD, particularly in the superior and inferior quadrants of the retina, compared to age-matched controls [2,37]. This suggests loss of retinal ganglion cell axons occurs in PD, in line with the broader neurodegenerative process [2,33]. Indeed, the toxic accumulation of α-synuclein protein—a pathological hallmark of PD—has been detected in retinal tissue of PD patients [2]. The presence of α-synuclein aggregates in the retina indicates that PD’s pathology extends beyond the brain, potentially contributing to retinal cell loss [2,33,37]. Correspondingly, some PD patients show visual symptoms (such as impaired color vision or contrast sensitivity) that correlate with these retinal changes [2].

Notably, retinal neurodegeneration in PD may manifest at very early stages of the disease or even precede classic brain lesions [25,37]. Experimental models provide striking evidence: in a rat model of PD induced by the mitochondrial toxin rotenone, researchers observed that retinal degeneration occurred before significant damage in the substantia nigra [37]. While the rotenone-induced rat model mimics mitochondrial dysfunction associated with Parkinson’s disease, its inherent retinal toxicity may not entirely reflect the complex pathophysiology seen in human cases. Within just 20 days of rotenone exposure, in vivo retinal imaging revealed increased apoptosis of retinal ganglion cells and edema (swelling) of retinal layers. In contrast, the hallmark nigral neurodegeneration was not yet fully developed [37]. These findings imply that the retina may exhibit neurodegenerative changes earlier than the deep brain structures in PD [25,37].

Clinically, this raises the exciting possibility of using retinal imaging to detect PD in its prodromal phase [2,25,37]. Early studies in humans have noted that certain retinal changes (such as thinning of specific inner retinal layers) correlate with disease severity and duration in PD patients [2]. While more research is needed, the current evidence supports the notion that PD entails a retinal pathology component—including RGC loss and protein aggregation—that can be monitored noninvasively [2,25,37]. This makes the retina a promising site for identifying early PD biomarkers and for tracking neuroprotective treatment effects as demonstrated in preclinical models (e.g., the neuroprotective agent rosiglitazone prevented retinal neuron loss in the rotenone PD model, preserving both eye and brain tissue) [37].

### 3.4. Retinal Imaging in Glaucoma and Other Neurodegenerative Conditions

Glaucoma is an optic neurodegenerative disease traditionally managed within ophthalmology, but it shares key features with CNS disorders [17,30]. In glaucoma, progressive death of retinal ganglion cells leads to optic nerve atrophy and characteristic vision loss [17,30,38,39]. It is well-established that OCT measurements of the retinal nerve fiber layer and ganglion cell–inner plexiform layer are sensitive biomarkers for glaucoma: affected individuals show significant thinning of these layers corresponding to visual field deficits [17,30,39,40]. Glaucoma has thus served as a model for studying neuroprotective strategies in the eye, and it exemplifies how retinal imaging can quantitatively track neurodegeneration [30,38,39]. In fact, some authors classify glaucoma as a chronic neurodegenerative disease of the eye with parallels to CNS disorders [30,38,39]. For instance, chronic glaucoma involves complex neurotoxic processes and even systemic risk factors, prompting comparisons with diseases like Alzheimer’s in terms of neurodegenerative mechanisms [30,38,39].

A cutting-edge retinal imaging approach in glaucoma is the Detection of Apoptosing Retinal Cells (DARC) technique [18,19,40]. DARC uses an intravenously administered fluorescent annexin V tracer that binds to phosphatidylserine on dying cells, combined with confocal scanning laser ophthalmoscopy, to visualize individual apoptotic retinal cells in vivo [18,19,40]. In glaucoma, RGC apoptosis is a precursor to irreversible vision loss, so detecting it early is crucial [18,19,40]. A first-in-human Phase I clinical trial of DARC demonstrated the feasibility and safety of this approach, showing that apoptotic retinal cells can be quantified in real time in patients [18,19,40]. The trial results suggested DARC could identify elevated rates of RGC death in glaucoma patients compared to healthy volunteers, indicating disease activity before extensive permanent damage occurs [18,19,40]. Thus, DARC imaging holds promise as an early in vivo indicator of neurodegeneration in glaucoma, potentially guiding timely intervention [18,19,40]. Beyond glaucoma, the DARC methodology is being explored in other conditions where retinal cell stress and loss are relevant [40]. For example, researchers have applied DARC to track retinal neurodegeneration in optic neuritis and age-related macular degeneration, and even used an AI-assisted DARC analysis to predict progression to wet AMD [40]. These applications underscore the broader relevance of apoptosis imaging as a biomarker for neurodegenerative changes in the retina [40].

Other novel retinal imaging biomarkers have also emerged. In glaucoma patients and suspects, studies using flavoprotein fluorescence at the optic nerve head have detected abnormal mitochondrial signals, reflecting metabolic dysfunction in optic nerve tissues [41,42]. This indicates that retinal imaging can capture metabolic stress in neurodegenerative eye disease [10,41,42]. Meanwhile, OCT angiography has been applied to neurodegenerative disorders beyond MS and AD [13,18,29,32,43]. For instance, reduced retinal perfusion and capillary density have been reported not only in glaucoma but also in some patients with AD and PD, aligning with the shared microvascular pathology seen in those brain disorders [29,32,43]. A recent review of retinal OCT-A in neurodegenerative diseases concluded that the retinal microcirculation shows parallel features to cerebral microcirculation in these conditions, and microvascular changes observed via OCT-A could serve as noninvasive proxies for neurovascular degeneration in the brain [32,39]. Table 1 provides a summary of key retinal imaging findings and potential biomarkers in major neurodegenerative diseases.

Advances in imaging technology have enabled researchers to visualize the retina at near-cellular resolution and to monitor physiologic signals [20,21,44,45]. Adaptive optics (AO) retinal imaging is one such innovation; by correcting optical aberrations, AO allows novel detail [20,24,44,45,46]. For example, AO combined with two-photon laser scanning has achieved nearly diffraction-limited imaging of retinal microstructures in vivo [20,21,22,24,47]. Using AO two-photon microscopy, investigators have observed real-time dynamics of retinal microglia and neurons in animal models of disease [20,22,47]. These high-resolution modalities have even been used to simultaneously capture neuronal activity and blood flow in the retina, providing an integrative view of neurovascular interactions [20,22,27,47]. Similarly, hyperspectral retinal imaging has been piloted in mouse models of Parkinson’s and in aging, aiming to detect subtle spectral signatures of retinal pathology [25]. While these experimental techniques are not yet in routine clinical use, they represent the future of retinal biomarkers—moving beyond mere anatomy to capture cell-specific dysfunction and physiology in neurodegenerative diseases [2,20,22,24,25,27,44,45].

## 4. Discussion

The findings of this review reinforce the principle that the eye can serve as a sensitive barometer of neurodegenerative processes in the brain. Across multiple studies and modalities, the retina has been shown to reflect pathologies in diseases such as MS, glaucoma, AD, and PD, lending credence to the concept of the retina as an accessible “window” into the CNS [1,25,28]. This aligns with prior observations that retinal changes—whether structural thinning, cellular loss, or microvascular alterations—often parallel those occurring in the optic nerve and brain [14,25,26]. At the same time, our analysis highlights that each photonic imaging modality interrogates a different aspect of retinal biology, underlining the complementary nature of these tools. By contextualizing our results with earlier literature, we discuss below how structural, physiologic, vascular, and metabolic imaging findings converge or diverge, the strengths and limitations of each modality, and the implications for various neuro-ophthalmologic diseases.

### 4.1. Structural vs. Functional Imaging Modalities

A key theme is the distinction between structural imaging techniques (which capture anatomical changes like neuronal loss or tissue thickness) and physiologic or metabolic imaging techniques (which capture dynamic processes or physiological states). OCT exemplifies the structural approach, providing quantitative measures of retinal nerve fiber layer (RNFL) thickness and ganglion cell layer integrity. In both MS and glaucoma, OCT-detected thinning of these retinal layers is a well-established surrogate for neuroaxonal loss, correlating with clinical dysfunction and disease burden [2,10,13,14,17]. Such structural biomarkers are highly reproducible and already widely used in practice; for instance, serial OCT can monitor progressive RNFL loss in patients with MS or glaucoma, serving as an objective measure of neurodegeneration over time [13,14,16].

However, purely structural OCT metrics primarily reflect cumulative neuroaxonal loss and may not fully capture short-term or early disease activity. In practice, structural progression can only be identified once the observed change exceeds measurement variability, so early or focal activity may remain below the threshold of reliable detection—particularly when loss is diffuse [15,16,48]. Accordingly, complementary cellular and metabolic approaches can help bridge this gap by detecting earlier signs of neuronal stress or ongoing cell death when thickness metrics remain within variability limits [18,19].

Emerging cellular, physiologic, and metabolic modalities directly visualize aspects of retinal physiology and pathology that precede gross structural loss. The Detection of Apoptosing Retinal Cells (DARC) technique is one such example, using fluorescently labeled probes to identify retinal ganglion cells undergoing apoptosis in vivo [18,19]. DARC provides real-time insight into neuronal death: studies have demonstrated that individual dying cells can be imaged in patients with glaucoma before widespread RNFL thinning occurs [18]. This represents a paradigm shift—instead of inferring neural damage after the fact (as OCT does), DARC allows us to observe neurodegeneration as it unfolds. Likewise, fluorescence lifetime imaging ophthalmoscopy (FLIO) and hyperspectral retinal imaging offer physiologic and metabolic information by measuring, respectively, the decay kinetics of intrinsic retinal fluorophores and the spectral signatures of retinal tissues. These techniques can reveal abnormalities in retinal metabolism or composition (for example, altered fluorophore lifetimes or reflectance spectra) that may correlate with early neurodegenerative changes [25,26].

FLIO has shown promise in detecting metabolic deficits in the retina that correspond to brain pathology in diseases affecting vision, while hyperspectral imaging has identified distinctive retinal spectral patterns in neurodegenerative disease models [25,26]. Thus, functional imaging modalities add a critical dimension, often detecting pathology at a stage when structural changes are subtle or absent. They complement structural tools by providing a more immediate readout of neuronal function, cell viability, or metabolic state [18,26]. It is noteworthy, however, that these advanced techniques remain largely investigational; issues such as signal specificity, the need for specialized equipment, and interpretive complexity must be addressed before they can transition to routine clinical use [21,25]. In summary, structural, cellular, physiologic, and metabolic retinal imaging should be viewed as complementary rather than competitive approaches: the former excels at quantifying established damage in a clinically accessible way, while the latter aspires to identify incipient or ongoing pathology, potentially enabling earlier intervention [19,26]. Table 2. provides an overview of emerging photonic imaging modalities in neuro-ophthalmology, the primary mechanisms, and key applications.

### 4.2. Comparison of Imaging Modalities and Their Complementary Strengths

Each photonic imaging modality reviewed brings distinct strengths and suffers from its own limitations. A comparative look at our findings illustrates how these technologies can complement each other in illuminating the eye–brain connection. OCT and its variant, OCT-angiography (OCT-A), provide high-resolution, depth-resolved structural maps of the retina and optic nerve head. Their strengths lie in precision and accessibility: OCT is noninvasive, rapid, and quantitatively reliable, making it a mainstay for both clinical monitoring and research [13,14]. In neuro-ophthalmology, OCT’s ability to measure retinal layer thickness has translated into concrete biomarkers (e.g., ganglion cell layer and RNFL thickness) that correlate with disease severity and progression in conditions such as MS and glaucoma [9,10,12,17]. OCT-A extends this by visualizing the retinal microvasculature without dye injection, thereby capturing the microcirculatory aspect of neurodegeneration. Notably, our review underscored that retinal capillary density changes on OCT-A mirror cerebral microvascular pathology in neurodegenerative diseases such as AD and PD, suggesting that retinal microvascular health is a proxy for brain microvascular health [29,32]. However, OCT and OCT-A are fundamentally anatomical/structural imaging; they do not directly report on cell metabolism or function. Small physiologic alterations may go unnoticed until they manifest as structural damage or perfusion deficits. Moreover, artifacts and inter-patient anatomical variability (e.g., variations in optic nerve head dimensions or baseline RNFL thickness) can complicate cross-sectional comparisons [34,39]. Despite these caveats, the breadth of evidence supporting OCT/A in diverse settings—from MS clinics to glaucoma screening—affirms it as an indispensable baseline tool [14,17]. Any new modality will likely augment rather than replace its role.

Adaptive optics (AO) imaging and two-photon laser scanning microscopy represent the frontier of resolution in retinal imaging. By correcting optical aberrations of the eye, AO allows existing imaging modalities to resolve microscopic structures that were previously invisible in vivo [20,22,44]. When AO is paired with two-photon excitation microscopy, researchers can achieve nearly diffraction-limited views of retinal neurons, glia, and capillaries in living eyes [20,24]. Our synthesis of recent studies found that AO-assisted two-photon imaging has, for the first time, enabled subcellular visualization of retinal architecture in animal models—from the fine dendritic arbors of ganglion cells to the dynamic behavior of microglial cells surveying the retinal milieu [21,22]. Such ultra-high resolution is exceptional in human neuroscience; it lets us watch pathological processes (like microglial activation, inflammatory cell trafficking, or dendritic pruning) unfold in real time within retinal tissue [21,22].

The obvious strength here is insight: these tools reveal mechanistic details and spatiotemporal dynamics that bridge the gap between microscopic pathology and clinical imaging. For example, observations of hyperactive retinal microglia in diabetic mouse models, occurring well before overt vascular damage, provide a vivid parallel to early neuroinflammatory changes hypothesized to occur in diabetic retinopathy and possibly in neurodegenerative disorders [22]. Similarly, investigators have simultaneously imaged neuronal calcium signals (activity) and capillary blood flow in the retina, demonstrating neurovascular coupling at a microscopic scale [27,47]. These feats underscore how photonic imaging can explore retinal neurobiology in ways analogous to two-photon microscopy in the brain, but through the transparent ocular media rather than through a skull.

The limitation, of course, is that AO and two-photon systems are technologically demanding. They are largely confined to research laboratories; two-photon ophthalmoscopy in humans is in an early feasibility stage and involves expensive femtosecond lasers and stringent safety controls [23,24]. Field of view is also limited—one can image a small patch of the retina at high magnification, rather than the wide fields that clinical OCT can cover. Moreover, translating this resolution to clinical value is not straightforward: capturing high-resolution images of a single cell is technologically impressive, but we must determine how such granular information can inform patient care or diagnostics in practice. Thus, while AO and two-photon modalities push the boundaries of what can be seen (offering a “microscope” view of neurodegeneration in the eye), their current role is primarily to advance understanding of disease mechanisms rather than to serve as routine diagnostics. Over time, technical refinements and automation may bring some of these capabilities to the clinic, perhaps in the form of high-resolution retinal cameras that can reliably detect cellular-level changes [45]. Until then, they remain powerful research tools that complement population-level clinical imaging by validating and discovering retinal biomarkers at the single-cell level.

Cellular, physiologic, and metabolic imaging modalities add yet another layer of complementary information. We have already discussed DARC, which directly visualizes apoptotic cells. Its strength is temporal immediacy—detecting neural injury as it occurs—making it potentially invaluable for conditions like glaucoma, where intervention timing is critical [18]. Still, DARC requires an intravenous tracer and specialized confocal imaging, factors that currently limit it to clinical trials. The specificity of DARC signals (distinguishing pathological apoptosis from normal cell turnover) and its applicability beyond optic neuropathies (e.g., in detecting neuronal death in MS or AD retinas) remain active research questions [19]. FLIO, on the other hand, is completely non-invasive and leverages natural retinal fluorophores (such as metabolic cofactors or lipofuscin) to infer tissue metabolism. By measuring fluorescence lifetimes in retinal tissues, FLIO can identify subtle metabolic perturbations in diseases—for example, prolonged lifetimes might indicate accumulation of metabolic byproducts or impaired energy metabolism in retinal cells [26]. One of the reviewed studies linked abnormal retinal FLIO readings to cortical changes, suggesting that retinal metabolic dysfunction might parallel brain pathology in demyelinating or degenerative disorders [26]. The ability of FLIO to detect changes even when structural imaging is normal is a key advantage; it might flag patients who are at risk or in prodromal stages of disease. Hyperspectral imaging is also a label-free method that captures a spectral fingerprint of retinal tissue. Our review noted its application in Parkinson’s disease models: investigators detected a unique retinal reflectance signature in PD, distinct from that of normal aging, that could relate to changes in melanin, mitochondrial function, or other molecular alterations in the PD retina [25]. In Alzheimer’s disease, hyperspectral retinal imaging has been investigated as a non-invasive approach to detect retinal spectral signatures associated with cerebral amyloid burden, although this strategy remains preliminary and requires further validation [49].

These techniques collectively shift the focus from morphology to chemistry and cell physiology, expanding the diagnostic palette. The main drawback, however, is that these imaging modalities produce complex datasets that require advanced analytical methods for interpretation, and their clinical validation is still in the early stages. Small sample sizes and prototype instrumentation are common in these studies, so results must be confirmed in larger longitudinal trials [34]. Additionally, not all physiologic or metabolic changes detected may be specific to a given disease. For instance, a metabolic abnormality on FLIO could arise from various retinal conditions, not just a neurodegenerative process. Therefore, rigorous standardization and a clearer understanding of normal versus disease-related spectroscopic ranges will be needed before broader clinical translation. In sum, by combining structural imaging (to map the “where” and “how much” of tissue loss) with cellular, physiologic, and metabolic imaging (to understand the “how” and “why” of cellular dysfunction), we get a far richer picture of the neuro-ophthalmic landscape than either alone could provide. The complementary strengths of these modalities underscore a likely future in which multimodal retinal imaging is employed, harnessing each technique’s advantages to achieve sensitive and specific detection of neurological disease activity [27,39].

### 4.3. Disease-Specific Implications and Translational Relevance

Multiple Sclerosis (MS): The discussion of our results in the context of MS illustrates a strong consensus in the literature: the retina is an important site of neurodegeneration in MS and can be quantitatively assessed for insights into the disease. Numerous studies have shown that MS patients exhibit thinning of the peripapillary RNFL and ganglion cell/inner plexiform layer on OCT, even outside of acute optic neuritis episodes [2,10,13,14]. This retinal atrophy correlates with optic nerve damage and disability. For example, greater OCT thinning is associated with worse visual outcomes and higher MS lesion load or brain atrophy on MRI [10,12]. Our review confirmed these relationships, reinforcing that OCT-derived metrics are robust biomarkers for MS progression [12,14]. In fact, several longitudinal studies and meta-analyses have endorsed the use of OCT in MS to track neuroprotective treatment effects and long-term neuronal loss, leading to proposals that OCT outcomes be incorporated into clinical trials and routine monitoring of MS patients [12,13,14,15]. The translational relevance is clear: OCT provides a window into the CNS that is quicker and less expensive than MRI, and it can detect diffuse neuroaxonal loss that might otherwise go unrecognized in early or mild disease [14,15]. However, areas of divergence remain.

Some studies note that while group-level differences in retinal thickness between MS patients and controls are reproducible, the sensitivity for individual patient monitoring can be variable [34]. Inter-eye and inter-subject variability, as well as contributions from normal aging, can complicate the interpretation of small changes over time in an MS patient’s OCT measurements. Additionally, OCT largely captures the end result of inflammation (axonal loss) rather than active inflammation itself. Here, complementary modalities might help: although not yet routine in MS, experimental approaches such as DARC or FLIO could, in principle, identify ongoing neurodegenerative processes in MS.

For instance, an increase in apoptotic retinal cells or a shift in retinal metabolic fingerprints might signal active disease even when OCT thickness is relatively preserved. While our review did not find extensive MS-specific applications of DARC or FLIO in humans, it highlighted this conceptual possibility. Indeed, one study using FLIO suggested retinal metabolic anomalies in MS that correlated with MRI measures of cortical damage, hinting that retinal metabolism can reflect widespread CNS injury in MS [26]. Further research is needed to validate such findings and determine whether cellular or metabolic retinal imaging could predict MS disease activity or progression before structural loss accumulates. In multiple sclerosis, reduced retinal vessel density should therefore be interpreted cautiously, as it may reflect secondary vascular remodeling driven by chronic neuroaxonal degeneration, subclinical optic pathway involvement, or reduced metabolic demand, rather than a primary retinal vascular target of the disease [13,14,32]. Mechanistic conclusions should therefore not be drawn from correlations between vessel density and disease severity alone, unless supported by longitudinal, experimental, or interventional evidence capable of clarifying temporal and causal relationships [31,32,34].

Glaucoma: As a quintessential neuro-ophthalmic disorder with a primary ocular focus, glaucoma demonstrates how ophthalmic imaging modalities contribute to both research and clinical care. Our findings support the well-established role of OCT in glaucoma: thinning of the RNFL and ganglion cell complex on OCT is a hallmark of glaucomatous damage and is used daily in practice to diagnose early glaucoma and monitor its progression [13]. This structural change corresponds to the loss of retinal ganglion cells, making OCT a surrogate for neuronal loss in the optic nerve. However, because glaucoma may silently progress until a significant fraction of cells are lost, cell death and physiologic imaging approaches have garnered interest as methods to detect active disease.

The DARC technique emerged specifically from this need. In early-phase clinical studies, DARC has enabled direct visualization of retinal ganglion cells undergoing apoptosis in glaucoma patients, thereby capturing cell death events that occur months or years before enough cells die to appreciably thin the RNFL [18].

Cordeiro et al. reported higher DARC counts in glaucoma patients with documented progression than in healthy controls, supporting the ability of DARC to capture ongoing apoptotic activity in vivo [18]. In a post hoc exploratory analysis, higher DARC signals were associated with faster subsequent progression relative to baseline; however, the sample size was small, and these findings should be interpreted cautiously [18]. A subsequent study in a larger cohort provided suggestive evidence that elevated DARC values may have prognostic value for future progression, although confirmation in adequately powered trials is still required [19]. Our review places these findings in context: while DARC’s promise is evident—offering a real-time cell-death biomarker of disease activity—it is still investigational. Questions about its specificity (do other retinal conditions also show DARC spots?), optimal quantification methods, and long-term safety will need to be resolved before DARC can transition to the clinic.

Another emerging approach for glaucoma is the use of OCT-A to assess retinal microcirculation; some studies suggest reduced perfusion in the optic nerve head and superficial peripapillary retinal plexus in glaucoma, reflecting the vascular component of the disease, although interpretation is intricate [43,50]. Our synthesis indicates that combining OCT (structure), OCT-A (microvascular function), and possibly DARC (cellular function) could provide a multidimensional view of glaucoma—distinguishing patients whose disease is active (ongoing cell loss identifiable by DARC) from those whose disease is stable (no apoptotic signal, exhibiting a stable structural loss over time, devoid of indications of continued progression). This kind of comprehensive retinal profiling is not yet routine, but it represents a future direction for personalized glaucoma management.

Beyond purely structural and vascular metrics, there is growing interest in complementary electrophysiologic and metabolic approaches that may refine glaucoma phenotyping. Pattern electroretinography has shown potential for detecting early retinal ganglion cell dysfunction before marked structural thinning becomes evident, and studies combining PERG with OCT angiography suggest that reduced electrophysiological responses may parallel macular or peripapillary vascular compromise in normal-tension glaucoma [51,52]. Mitochondrial imaging and flavoprotein fluorescence have also highlighted metabolic stress at the optic nerve head in glaucoma, supporting the view that retinal imaging can capture physiologic impairment in addition to structural loss [41,42]. At present, however, the strongest evidence in glaucoma still supports OCT and OCT-A for structural and microvascular assessment, with DARC remaining a promising but investigational method for detecting active apoptotic activity at an earlier stage [17,18,19,43,50]. Electrophysiological findings also support the idea that physiologic impairment in glaucoma may extend beyond purely structural retinal loss, although these approaches remain complementary to the core retinal imaging framework reviewed here [53].

While glaucoma is increasingly discussed in the context of neurodegeneration, the interpretation of retinal and central nervous system changes should be done with caution. Many of the structural, vascular and metabolic abnormalities reported in the retina and visual pathway may be secondary to primary retinal ganglion cell injury at the optic nerve head rather than evidence of a separate generalized neurodegenerative disorder [17,30,39]. Experimental and clinical studies show that loss of retinal ganglion cells is followed by trans-synaptic changes in the visual pathway, including the lateral geniculate nucleus and visual cortex [30,39]. Thus, some of the reported central nervous system imaging findings in glaucoma are not indicative of primary brain disease but rather secondary degeneration. Multiple mechanistic similarities exist between glaucoma and neurodegenerative diseases, including mitochondrial dysfunction, neuroinflammation, axonal transport impairment and progressive neuronal degeneration [30,39,41,42]. However, whether glaucoma should be considered as a neurodegenerative disease outside the eye is still controversial.

Alzheimer’s Disease (AD): Among neurodegenerative diseases, AD presents both an exciting and challenging frontier for retinal imaging. The premise driving research is that Alzheimer’s pathology in the brain (amyloid plaques, tau tangles, neurodegeneration) might be detectable in the retina, given embryological links and the retina’s CNS nature. Our review documented several structural retinal changes reported in AD: for instance, thinning of the inner retinal layers (RNFL and ganglion cell layer) has been observed in AD patients compared to age-matched controls, in line with the loss of CNS neurons. Some investigators have found that retinal thinning or volume loss correlates with the severity of cognitive impairment or with biomarkers of AD (such as brain amyloid load on PET imaging), suggesting that the retina may mirror disease progression [2,34].

Vascular changes have also been noted: using OCT-A, researchers have identified reduced retinal capillary density or perfusion in AD, which parallels cerebral microvascular compromise often seen in AD brains. In addition, retinal oximetry has been explored in AD, suggesting altered retinal oxygenation metrics [29,32]. These findings collectively support the concept of retinal biomarkers for AD and align with the broader notion of small vessel involvement and neurodegeneration in the disease. However, the literature in this area is not entirely uniform. Some studies have failed to find significant retinal layer differences in AD, or have found that any differences are confounded by age-related changes and ocular comorbidities [2,34]. Divergence arises from differences in imaging protocols, sample sizes, and disease stages across study cohorts. For example, mild cognitive impairment or early AD might show very subtle retinal changes, requiring larger sample sizes to detect. In contrast, moderate-to-advanced AD might have more pronounced thinning—but by that stage, comorbid retinal aging changes may muddy the picture. Our results acknowledge this uncertainty; while the trend in the evidence favors retinal changes in AD, it is clear that more standardized methods and longitudinal data are required in order to confirm their reliability and specificity [32,34].

Beyond structural metrics, AD research has spurred some of the most innovative applications of photonic imaging in the eye. Notably, attempts to directly visualize molecular hallmarks of AD in the retina have been reported. Investigators have explored tracer-based and label-free optical approaches to probe molecular hallmarks of AD in the retina [2,34]. Retinal hyperspectral imaging has been investigated as a non-invasive approach to identify spectral signatures associated with cerebral amyloid burden, raising the possibility of an optical adjunct screening strategy; however, validation remains preliminary [49]. Similarly, pilot studies using fluorescence lifetime imaging ophthalmoscopy (FLIO) have reported altered retinal fluorescence lifetimes in preclinical/early Alzheimer’s disease, consistent with metabolic changes, although specificity for amyloid pathology is not yet established [54]. Overall, these emerging modalities remain in an early phase and require larger longitudinal studies and standardization before clinical translation. These cutting-edge approaches underscore the retina’s potential as a surrogate for brain pathology—essentially, an accessible screening site for diseases like AD that currently require costly or invasive tests (e.g., PET scans or CSF analysis). The limitations are evident, however. Signal specificity is a major concern: distinguishing amyloid-specific signals from other age-related deposits (like lipofuscin or drusen in the retina) is challenging, and false positives or negatives could occur.

Additionally, the required technology (advanced hyperspectral cameras, scanning laser ophthalmoscopes with fluorescence lifetime capabilities) is not widely available outside research settings. We emphasize that while early results are promising, there is a need for large-scale studies to validate these retinal imaging biomarkers against gold-standard brain biomarkers for AD, and to determine their predictive value for conversion from asymptomatic to symptomatic stages [2,34]. In terms of translational outlook, the field largely agrees on the tantalizing opportunity retinal imaging presents for AD diagnosis and monitoring, but diverges on how close we are to realizing that in clinical practice. With continued research, it is conceivable that an ophthalmic exam could one day contribute to AD risk stratification—for example, by identifying individuals with retinal signs of amyloid who may benefit from early therapeutic interventions—but this remains a future goal pending further evidence [31,32].

Parkinson’s Disease (PD): PD is another systemic neurodegenerative disorder where retinal imaging findings are being actively explored. Dopaminergic amacrine cells in the retina and the neural circuits for vision can be affected by PD, raising the question of whether retinal changes could serve as biomarkers for the disease. The studies in our review and prior literature have reported several retinal alterations in PD patients: thinning of the peripapillary RNFL and inner retinal layers has been observed in some PD cohorts, albeit not as consistently as in MS or AD [2,37]. These structural changes, when present, are hypothesized to result from trans-synaptic degeneration (loss of retinal cells secondary to brain dopaminergic neuron loss) or direct involvement of ocular neurons by the disease process.

Some OCT-A studies in PD have also noted reduced vessel density in the superficial retinal capillary plexus, potentially reflecting autonomic or microvascular dysfunction associated with PD [2,32]. However, results vary, and not all studies detect significant OCT differences in PD, especially in early disease or small samples [2,32]. A notable point of agreement is that any retinal changes in PD are subtle—much subtler than in optic neuropathies—and thus advanced imaging or larger studies are needed to validate them. This is where emerging photonic modalities, such as hyperspectral imaging, have made their mark. One compelling finding highlighted in our review is the identification of a PD-specific spectral signature in the retina using hyperspectral imaging [25]. In mouse models of PD, HSI revealed distinct retinal reflectance patterns compared to healthy controls [25]. Importantly, an initial clinical study found that PD patients also exhibit altered retinal reflectance spectra—in particular, a reduction in short-wavelength (blue) reflectance—relative to age-matched controls. These spectral changes might reflect alterations in retinal pigment or metabolism associated with PD pathology (for example, changes involving melanin/neuromelanin or oxidative stress markers in the retina). If reproducible, hyperspectral retinal imaging could become a non-invasive screening tool for PD or a means to monitor disease progression by analyzing the retina’s spectral “fingerprint”.

Another interesting avenue is physiologic imaging: since dopamine is crucial for normal retinal function (e.g., contrast sensitivity and adaptation), PD may cause vision-related physiologic changes that could be detectable with electroretinal techniques or OCT-based physiologic metrics, even if overt structural changes are minimal. While our review did not delve into electroretinography, prior studies have shown altered visual processing in PD (e.g., impaired contrast sensitivity or color discrimination), which indirectly supports the notion that the retina and visual pathways are affected [2].

Translationally, retinal imaging for PD remains in an exploratory phase. There is enthusiasm that the eye exam might assist in early PD detection, especially since motor symptoms appear only after significant neurodegeneration has occurred systemically. For example, if a hyperspectral imaging device could flag individuals with an “at-risk” retinal spectrum for PD (perhaps even before motor onset), it could lead to earlier neurologic evaluation and intervention. This remains speculative but is the kind of future direction being actively researched.

A challenge specific to PD is the need to differentiate PD retinal changes from those due to normal aging or other eye diseases common in the elderly. Careful study design and multimodal imaging approaches, combining structural OCT with physiologic or hyperspectral assessments, will be required to improve specificity in PD. Future longitudinal studies should determine whether retinal changes can reliably serve as surrogate markers of CNS pathology in Parkinson’s disease [2,25,55]. Our discussion of PD underscores a broader theme: the retina as a site of early or prodromal signs. Just as hyposmia and REM sleep behavior disorder are early clues for PD, subtle retinal abnormalities might join this list of preclinical markers if validated. This is an area ripe for future longitudinal studies and technological innovation.

### 4.4. Complementarity of Modalities and Future Directions

An overarching insight from this review is that no single imaging modality is sufficient to capture the full spectrum of neuro-ophthalmic changes in complex diseases. Rather, each modality provides one piece of the puzzle. There is growing recognition that integrating multiple retinal imaging techniques—structural, physiologic, and metabolic—yields a more robust assessment than any alone [19,27,39]. For instance, in MS, one might use OCT to establish baseline neuroaxonal loss, OCT-A to evaluate the microvascular health of the retina, and an emerging modality (like FLIO) to probe metabolic status.

In glaucoma, OCT can monitor chronic structural loss, DARC detects acute apoptotic events, and OCT-A evaluates the existence of circulating blood in retinal vessels rather than total perfusion, offering a comprehensive picture of disease activity. In AD and PD, combining structural measures (to quantify degeneration) with hyperspectral or FLIO data (to detect molecular signatures) could significantly improve diagnostic accuracy and our understanding of disease mechanisms. Such multimodal approaches are already being explored in AD and PD research settings and represent a logical evolution of the field [26,49,54,55]. They also echo trends in neurology, where combinations of biomarkers (imaging, CSF, genetic) are used to enhance diagnostic confidence and track disease.

From a translational standpoint, one must consider differences between modalities that are clinic-ready versus those still in the laboratory or early trial phase. OCT and OCT-A are firmly in the clinical realm; they are non-invasive, relatively affordable, and fast, with established reference data and standardized platforms [14,17,39]. In contrast, technologies like AO-two-photon microscopy, DARC, FLIO, and hyperspectral imaging are currently used in limited research contexts or clinical studies. Bringing these advanced modalities to the clinic will require overcoming practical hurdles. For DARC, as noted, it means validating an injectable tracer’s safety and efficacy in larger glaucoma (and possibly MS or AD) trials, and demonstrating that early detection using DARC leads to improved patient outcomes (for example, by enabling earlier treatment adjustments) [18,19].

For two-photon and AO imaging, it means engineering more compact and user-friendly devices—perhaps leveraging eye-tracking and automated aberration correction—so that a high-resolution “retinal microscope” could be operated in a clinical office setting without specialist intervention [23,24,45]. FLIO and hyperspectral systems will need rigorous standardization of data acquisition and analysis; currently, different research groups may employ different excitation sources or analysis algorithms, making it hard to compare results across studies. To address this, consensus efforts are likely needed to establish protocols, much as has been done for OCT (e.g., guidelines on segmentation and quality control in OCT for MS and other diseases) [15].

Encouragingly, the trajectory of these technologies shows that translation is feasible—many began as lab prototypes and have steadily advanced (for example, OCT itself was once a laboratory tool in the 1990s before becoming ubiquitous by the 2010s). As computational power and optical engineering continue to improve, it is reasonable to expect that today’s experimental retinal imaging methods could be streamlined for broader clinical use in the coming decade [32,45].

Another aspect worth discussing is the agreement and divergence in how researchers interpret retinal changes relative to brain pathology. There is broad agreement that the retina often reflects CNS changes: this underpins the entire field of retinal biomarkers and is supported by numerous cross-sectional and longitudinal studies in MS, glaucoma, and even AD [1,29]. For example, in MS, virtually all studies agree that optic nerve inflammation and axonal loss leave a retinal imprint (thinning) that correlates with central disease [2,9,10,12,13,14]. Divergence emerges more in diseases where the eye findings are subtler or where the pathophysiological link is indirect.

In AD, while many studies report retinal changes, a few have not, leading to a debate on how reliable the retina is as a mirror for AD pathology [2,34]. Similarly, in PD, the magnitude of retinal changes is small, and studies are sometimes conflicting on their significance [2]. Some authors urge caution against over-interpreting retinal findings: just because the retina is an extension of the brain does not guarantee it will show all the same pathology present in the brain [2]. The retina might be affected by systemic neurodegeneration, but also has unique features (for instance, the retina lacks myelin except on the optic nerve fibers, it has a blood–retina barrier akin to the blood–brain barrier, but also local immune privilege) that could cause differences in how diseases manifest in the eye versus the brain. Our discussion recognizes this nuance. It is important to avoid a deterministic view that every neurodegenerative disease must have a retinal signature—instead, the retina provides a convenient window where changes often, but not always, parallel brain changes.

Recognizing areas of discordance (e.g., cases where brain pathology is significant but retinal changes are minimal, or vice versa) is crucial for refining our models of eye–brain relationships and for improving diagnostic specificity. Such discrepancies highlight the need for further research, particularly longitudinal studies that track patients over time to see if retinal imaging can predict outcomes or if certain subgroups show stronger eye–brain correlations than others [32,34]. They also point to the importance of multimodal confirmation: if a finding is truly reflective of CNS disease, it should correlate not just with one modality (say, OCT thickness) but with others (like MRI measures, clinical status, or fluid biomarkers). Future studies that incorporate a range of biomarkers will help clarify the retina’s role and reliability in each condition.

In light of the rapid advances documented, several future directions emerge organically from our review. One is the pursuit of earlier diagnosis: using retinal imaging to identify neurodegenerative diseases at a prodromal stage. This could involve screening at-risk populations (for example, family members of AD or PD patients, or individuals with genetic predispositions) with sensitive retinal imaging to detect early signs of pathology. Another direction is therapeutic monitoring: employing retinal biomarkers as outcome measures in clinical trials for neuroprotective drugs. Already, OCT is used as an endpoint in some MS trials to quantify neuroaxonal preservation; similarly, one could envision trials of AD treatments monitoring retinal amyloid plaque load via hyperspectral imaging or trials of PD neuroprotectants tracking retinal layer thickness or physiologic measures over time [15,31]. For such uses, consistency and standardization of retinal imaging techniques across centers will be paramount, which calls for consensus-building in the community (an effort that key reviews and expert panels are beginning to address) [31,42].

Additionally, the integration of artificial intelligence (AI) may greatly enhance the power of retinal imaging. AI algorithms can be trained to detect subtle patterns or combinations of features across modalities (for example, correlating OCT changes with hyperspectral data) that might be imperceptible to human observers. Applying machine learning to large retinal image datasets could improve disease classification and risk prediction, as has already been demonstrated in ophthalmology for diseases like diabetic retinopathy. While not a focus of our current review, this is an adjacent future research avenue that could accelerate the use of retinal imaging in neurology.

Finally, our review underscores the notion that the retina is not just a passive reflector of brain disease but can be an active contributor to our understanding of neuropathology. Retinal findings have prompted new hypotheses about disease mechanisms—for example, the pattern of retinal ganglion cell loss in AD might inform how neurodegeneration spreads, or the presence of retinal amyloid might provide clues to amyloid transport and clearance pathways [30,38]. Viewing the retina as an experimental model of the brain’s neurodegeneration is an exciting paradigm; it allows interventions (like neuroprotective drugs or gene therapies) to be tested in the eye with the possibility of directly observing outcomes at a cellular level (as with two-photon microscopy or DARC in animal models) [21,22]. This kind of translational research can then loop back to clinical care, informing strategies for early intervention or prevention.

In essence, the retina offers a unique convergence of accessibility and relevance: it is accessible enough to visualize microscopic processes in living patients, yet relevant enough to mirror critical aspects of CNS diseases. Our discussion, therefore, highlights an overarching agreement in the field—that advanced photonic retinal imaging is transforming neuro-ophthalmology by bridging disciplines and enabling discoveries that benefit both eye and brain science. Continued interdisciplinary collaboration and technological innovation will be key to fully realizing and standardizing the use of these emerging imaging modalities as reliable biomarkers and diagnostic tools for neurodegenerative diseases [31,32,45].

### 4.5. Limitations of Photonic Imaging Modalities

Despite the substantial progress outlined above, each photonic imaging modality retains important limitations that must be considered when interpreting its clinical or translational relevance. Structural approaches such as OCT are highly reproducible and clinically accessible, but they mainly detect established neuroaxonal loss and may be relatively insensitive to very early cellular dysfunction. In addition, segmentation artefacts, anatomical variability, and inter-device differences can complicate interpretation, particularly in longitudinal follow-up. Cellular, physiologic, and metabolic approaches introduce different challenges. DARC offers the possibility of detecting ongoing retinal ganglion cell apoptosis in vivo, but it still requires further validation with respect to disease specificity, quantification, reproducibility, and long-term clinical utility. FLIO and hyperspectral imaging provide potentially valuable information on retinal metabolism and molecular signatures, yet their outputs remain complex, are not fully disease-specific, and still depend on limited datasets and evolving analytical frameworks. Highly advanced modalities such as adaptive optics and two-photon imaging provide exceptional spatial resolution and mechanistic insight, but their current use is largely restricted to research settings because of cost, technical complexity, limited field of view, and restricted availability. More broadly, not all retinal alterations can be assumed to reflect central nervous system pathology in a direct one-to-one manner. Disease heterogeneity, ocular comorbidities, and biological differences between the retina and brain all affect interpretability. For these reasons, future progress in the field will likely depend on multimodal integration, standardization across platforms, and longitudinal validation in larger cohorts.

## 5. Conclusions

The evidence gathered in this review supports a clear paradigm shift: the retina is not merely an isolated ocular structure but an accessible extension of the central nervous system. For years, we have relied on OCT to quantify structural damage, and it remains a robust biomarker for tracking progression in diseases like multiple sclerosis and glaucoma [17,31]. However, waiting to detect structural atrophy often means intervening too late.

The real promise lies in the emerging ability to visualize pathology in real time. Techniques such as the Detection of Apoptosing Retinal Cells (DARC) are moving us from observing past damage to detecting active cellular death, offering a window for intervention before permanent vision loss occurs [18]. Similarly, by looking at metabolic changes through fluorescence lifetime imaging (FLIO) or hyperspectral analysis, we can now detect subtle molecular signatures that may parallel brain changes [25,26]. Even the vascular links are becoming clearer; OCT-angiography has shown that microvascular deficits in the eye often mirror the cerebral vessel pathology seen in Alzheimer’s and Parkinson’s disease [32,35]. Ultimately, the future of neuro-ophthalmology is not in a single tool, but in combining structural, cellular, physiologic, vascular, and metabolic perspectives. This multimodal approach has the potential to redefine how we diagnose and manage neurodegenerative conditions, shifting the focus from palliative care to early detection and preservation of neural function.

## Figures and Tables

**Table 1:** Retinal imaging biomarkers reported in major neurodegenerative and neuro-ophthalmic conditions (original compilation based on the cited literature).

Disease	Key Structural Findings (OCT/AO)	Physiologic & Vascular Findings (OCT-A/DARC/FLIO)	Eye–Brain Connection
Glaucoma	Significant thinning of RNFL and ganglion cell complex [17,30].	Real-time visualization of apoptosing RGCs using DARC [18,40]. Decreased superficial peripapillary capillary density on OCT-A [43].	Primary optic neuropathy characterized by retinal ganglion cell loss; extra-ocular visual pathway changes may partly reflect secondary trans-synaptic degeneration, while the extent of primary CNS involvement remains debated [30].
Alzheimer’s Disease (AD)	Thinning of pRNFL and ganglion cell–inner plexiform layer (GCIPL) reported on OCT in AD/MCI [33,34].	Reduced retinal capillary density on OCT-A [32,35]. Altered fluorophore lifetimes on FLIO suggesting mitochondrial dysfunction [26].	Retinal neurodegeneration and vascular changes may parallel cerebral pathology and cognitive decline [2,32,34].
Parkinson’s Disease (PD)	Inner retinal thinning (pRNFL) reported in some PD cohorts; preclinical models suggest early retinal neurodegeneration [2,37].	Distinct retinal reflectance signature on hyperspectral imaging in experimental and early human PD studies [25]. OCT-A studies report reduced superficial vessel density [32].	Retinal changes may appear early and could serve as prodromal markers [25,37].
Multiple Sclerosis (MS)	Thinning of pRNFL and GCIPL; correlates with disability (EDSS) and disease duration [9,12,31].	Reduced superficial capillary plexus density on OCT-A [9,28]. Potential metabolic anomalies on FLIO [26].	Retinal atrophy mirrors global CNS atrophy and can predict disability progression [2,31].

**Table 2:** Photonic imaging modalities in neuro-ophthalmology: targets, applications, and translational maturity.

Imaging Modality	Class	Primary Mechanism/Target	Representative Neuro-Ophthalmic Applications	Translational Status
Optical Coherence Tomography (OCT)	Structural	Depth-resolved interferometric imaging of retinal microstructure; layer segmentation and thickness metrics (e.g., RNFL, GCIPL).	Quantification of neuroaxonal loss and longitudinal monitoring in optic neuritis and MS; diagnosis and follow-up in glaucoma and other optic neuropathies.	Clinical Standard
OCT Angiography (OCT-A)	physiologic/Vascular	Motion-contrast OCT to map retinal and peripapillary microvasculature without exogenous dye; vessel density/perfusion metrics.	Assessment of retinal microvascular alterations in MS and other neurodegenerative disorders (e.g., AD, PD); adjunct vascular biomarkers in glaucoma.	Clinical/ Research
Detection of Apoptosing Retinal Cells (DARC)	Physiologic	In vivo fluorescence imaging of stressed/apoptotic retinal cells using an annexin-based tracer and confocal scanning laser ophthalmoscopy.	Early readout of active retinal ganglion cell death (particularly in glaucoma) to support earlier detection, risk stratification, and evaluation of neuroprotective strategies.	Clinical Trials/ Investigational
Adaptive Optics (AO) & Two-Photon Microscopy	Structural/ Physiologic	AO correction of ocular aberrations to approach diffraction-limited resolution; when combined with two-photon excitation, enables cellular/subcellular imaging and physiologic readouts mainly in experimental settings.	Mechanistic studies of retinal neurons, glia (including microglia), and microvascular dynamics in vivo; early human feasibility for two-photon excited retinal imaging.	Advanced Research
Fluorescence Lifetime Imaging Ophthalmoscopy (FLIO)	Metabolic	Time-resolved imaging of endogenous retinal fluorescence lifetimes as a proxy for metabolic state/oxidative stress.	Exploration of metabolic signatures associated with neurodegenerative disease (e.g., AD) and retinal disorders; potential adjunct biomarker for early dysfunction.	Research
Hyperspectral Retinal Imaging	Metabolic/ Structural	Acquisition of spatial–spectral retinal reflectance (or scattering) data to derive tissue-specific spectral fingerprints.	Investigation of disease-related spectral signatures in neurodegeneration (e.g., PD) and potential optical signatures linked to amyloid-related pathology in AD.	Research

## Data Availability

No new data were created or analyzed in this study. Data sharing is not applicable to this article.
